# Correction: Di Cesare Mannelli et al. Effects of the Combination of β-Hydroxy-β-Methyl Butyrate and R(+) Lipoic Acid in a Cellular Model of Sarcopenia. *Molecules* 2020, *25*, 2117

**DOI:** 10.3390/molecules31152585

**Published:** 2026-07-24

**Authors:** Lorenzo Di Cesare Mannelli, Laura Micheli, Elena Lucarini, Carmen Parisio, Alessandra Toti, Barbara Tenci, Matteo Zanardelli, Jacopo Junio Valerio Branca, Alessandra Pacini, Carla Ghelardini

**Affiliations:** 1 Department of Neurosciences, Psychology, Drug Research and Child Health-Neurofarba-Pharmacology and Toxicology Section, University of Florence. Viale Pieraccini 6, 50139 Florence, Italyalessandra.toti@unifi.it (A.T.);; 2 Department of Experimental and Clinical Medicine, Anatomy Section, University of Florence, Largo Brambilla 3, 50134 Florence, Italy; jacopojuniovalerio.branca@unifi.it (J.J.V.B.); alessandra.pacini@unifi.it (A.P.)

## Error in Figure

In the original publication [1], there was a mistake in Figure 4 as published. The corrected Figure 4 appears below (the legend of the figure was correct and has not been modified). The raw images are available upon request from the Editorial Office. The authors apologize for any inconvenience caused and state that the scientific conclusions are unaffected.

This correction was approved by the Academic Editor. The original publication has also been updated.

## Figures and Tables

**Figure 4:**
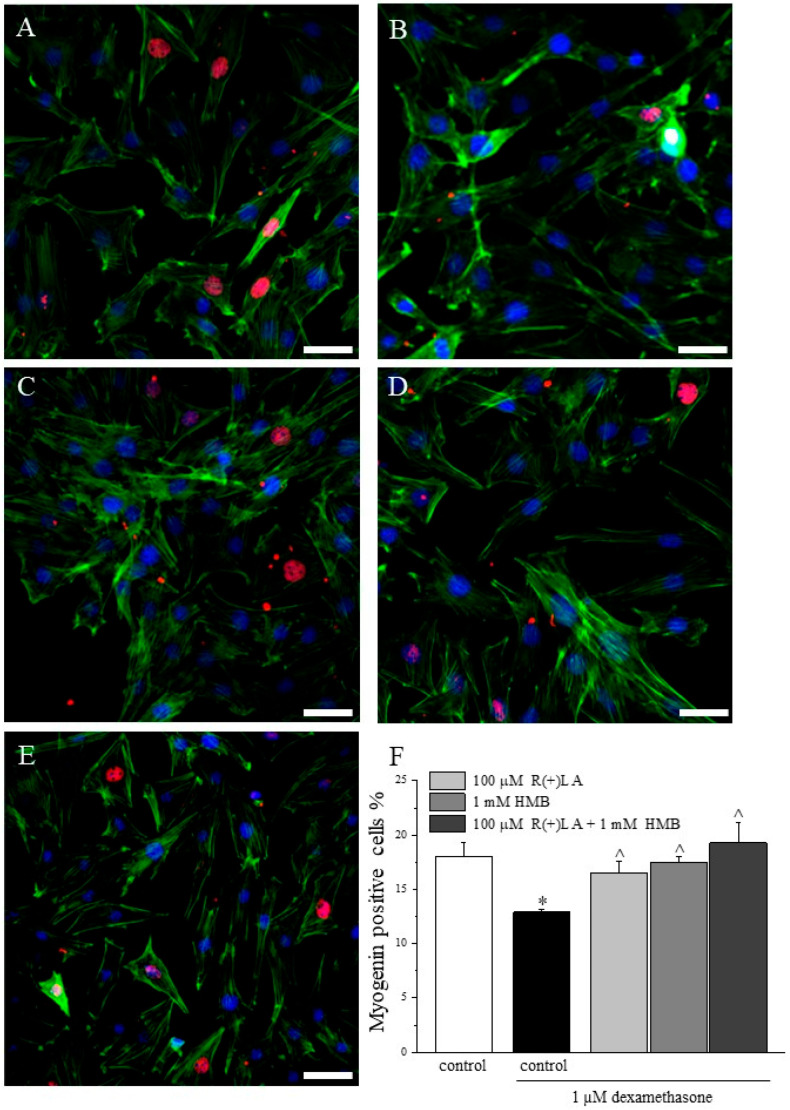
Myogenin staining of C2C12 cells after 72 h differentiation. C2C12 myoblasts were cultured for 24 h in differentiation medium (DM) and then incubated for 48 h as follows: with DM (**A**); with 1 μM DEX (**B**); with 1 μM DEX and 100 μM R(+)LA (**C**); with 1 μM DEX and 1 mM HMB (**D**); with 1 μM DEX, 100 μM R(+)LA and 1 mM HMB (**E**). The cells were then fixed and stained with Alexa488-conjugated phalloidin to evidence F-actin organization and a specific antibody that recognized myogenin (red). Nuclei were stained with 4′,6-Diamidine-2′-phenylindole dihydrochloride (DAPI) (blue). (**F**) Quantitative analysis of the percentage of myogenin-positive cells. The percentage was calculated by the ratio between the number of myogenin-positive cells (red) and the total cells identified by DAPI (100%). Scale bar (white line): 50 μm. Values are expressed as the mean ± S.E.M. of 3 different experiments. * *p* < 0.05 versus control (in the absence of DEX) and ^ *p* < 0.05 versus DEX treatment in the absence of R(+)LA and HMB.
