# Supplementary material for: Effects of the Combination of β-Hydroxy-β-Methyl Butyrate and R(+) Lipoic Acid in a Cellular Model of Sarcopenia
Source: Molecules. 2020 Apr 30;25(9):2117. doi: 10.3390/molecules25092117 (PMC7249096; doi:10.3390/molecules25092117)
Supplement: Supplementary file 1 [file molecules-25-02117-s001.pdf]

Supplementary Table S1. Viability of C2C12 myoblasts:

Cell viability %

	**dexamethasone concentration (µM)**			
	**0.01**	**0.03**	**0.1**	**10**
0	69.1 ± 1.8	62.4 ± 4.6	58.9 ± 4.3	52.3 ± 2.3
R(+)LA 1 µM	67.7 ± 1.7	63.8 ± 1.8	64.4 ± 5.5	54.5 ± 1.2
R(+)LA 10 µM	70.5 ± 2.0	58.6 ± 1.9	66.2 ± 7.3	57.9 ± 0.9
R(+)LA 30 µM	65.3 ± 2.9	57.0 ± 2.2	64.8 ± 6.0	57.2 ± 1.6
R(+)LA 100 µM	83.9 ± 3.6^	60.5 ± 1.1	71.3 ± 7.0	64.3 ± 1.3^
R(+)LA 300 µM	85.9 ± 4.9^	79.1 ± 6.6^	74.8 ± 8.0 #	74.1 ± 2.0^
HMB 30 µM	70.1 ± 1.5	72.7 ± 9.6	47.5 ± 6.6	49.1 ± 4.0
HMB 100 µM	71.8 ± 2.0	73.2 ± 9.2	49.8 ± 4.9	47.0 ± 3.2
HMB 300 µM	85.2 ± 3.8^	84.8 ± 9.4^	56.5 ± 6.0	44.5 ± 2.7
HMB 1 mM	97.9 ± 4.9^^	100.2 ± 10.9^^	69.7 ± 1.6	47.8 ± 3.5
HMB 3 mM	159.1 ± 6.7^^	108.5 ± 7.1^^	129.4 ± 2.8^^	52.4 ± 2.1

Cell viability of C2C12 myoblasts treated with increasing concentrations of dexamethasone (0.01 - 10 µM) in the absence or in the presence of increasing concentration of R(+)LA (1 – 300 µM) or HMB (30 µM – 3 mM) for 48 h. Cell viability was measured by MTT assay. Control condition was arbitrarily set as 100% and values are expressed as the mean ± S.E.M. of 3 experiments. ^P<0.05 and ^^P<0.01 *vs* the same treatment with dexamethasone in the absence of R(+)LA and HMB.

Supplementary Table S2. Viability of C2C12 myoblasts:

	cell viability %		
**concentration**	**R(+)LA**	**HMB**	**mixture**
0 μM	100 ± 2.4	100 ± 2.4	
1 μM	96.9 ± 1.2	98.3 ± 2.9	
10 μM	92.2 ± 2.1	100.3 ± 6.9	
30 μM	92.8 ± 3.1	90.8 ± 7.4	
100 μM	96.5 ± 5.1	93.2 ± 5.7	
300 μM	110.5 ± 3.3	92.6 ± 6.3	
1 mM		113.8 ± 3.1	
3 mM		194.7 ± 6.9**	
R(+)LA 30 μM + HMB 100 μM			106.2 ± 2.1
R(+)LA 30 μM + HMB 300 μM			125.0 ±2.6*
R(+)LA 100 μM + HMB 300 μM			128.0 ±1.6*
R(+)LA 100 μM + HMB 1 mM			169.4 ± 2.0*
R(+)LA 300 μM + HMB 3 mM			181.7 ± 4.1**

Cell viability of C2C12 myoblasts treated with increasing concentrations of R(+)LA (1-300 μM) or HMB (1 μM – 3 mM) or the combinations of both for 48 h. Control condition was arbitrarily set as 100% and values are expressed as the mean ± S.E.M. of 3 experiments. *P<0.05 and **P<0.01 *vs* control (0 μM).

Supplementary Table S3. Superoxide anion levels in C2C12 myoblasts:

($\mu M\ O_2^{\cdot -}$/mg protein/4h)	
**dexamethasone concentration (µM)**	**48 h incubation**
0	6178 ± 426
0.01	5877 ± 401
0.03	8328 ± 389*
0.1	9828 ± 549*
0.3	10596 ± 255*
1	11390 ± 562*
10	10980 ± 518*
100	12012 ± 445*

$O_2^{\cdot -}$ concentration of C2C12 myoblasts treated with increasing concentration of dexamethasone (0.01 - 100 µM) for 4 h. The non-specific absorbance was measured in the presence of SOD (300 mU/ml) and subtracted from the total value. Values are expressed as µM/mg protein/4h. Bars represent the mean ± S.E.M. of 3 experiments. *P<0.05 *vs* control (0 µM).
